# Genetic and Environmental Factors Contributing to the Pathogenesis of Vertebral Osteomyelitis Caused by *Enterococcus cecorum* in Broiler Chicken

**DOI:** 10.3390/ani15162327

**Published:** 2025-08-08

**Authors:** Ummu Syauqah Al Musyahadah, Andi Asnayanti, Anh Dang Trieu Do, Adnan Alrubaye

**Affiliations:** 1 Center of Excellence for Poultry Science, University of Arkansas, Fayetteville, AR 72701, USA; syauqah@uark.edu (U.S.A.M.); aasnayan@uark.edu (A.A.); ad086@uark.edu (A.D.T.D.); 2 Department of Poultry Science, University of Arkansas, Fayetteville, AR 72701, USA; 3 Bioinformatics Study Program, Faculty of Health Technology, University of Megarezky, Makassar 90234, Indonesia; 4 Cell and Molecular Biology Program, University of Arkansas, Fayetteville, AR 72701, USA

**Keywords:** vertebral osteomyelitis, *Enterococcus cecorum*, environment, genetics, broiler

## Abstract

*Enterococcus cecorum* (EC) is the primary cause of Vertebral Osteomyelitis (VO) in poultry, characterized by a hock-sitting posture with legs extended forward. The disease begins with intestinal colonization, where pathogenic strains of *E. cecorum* establish themselves in the gut within the first week of a bird’s lifecycle and translocate to the bloodstream later, reaching the organs, which finally infect the birds’ Free Thoracic Vertebrae (FTV). Several factors contribute to the initiation and progression of the disease. Environmental factors such as heat stress, high blood sugar levels, prior infections, overcrowding, and rapid growth conditions weaken immunity and stimulate the emergence of VO. Survivability of the pathogenic strains in the environment and the presence of virulence factors support its pathogenicity. A number of specialized virulence factors are known to be present in pathogenic *E. cecorum*, supported by the presence of potential mobile elements and resistance genes, which are also mainly present in commensal strains.

## Simple Summary

Environmental and genetic factors are substantial external and internal factors, respectively, that contribute to the development of pathogen infections. This review provides an overview of the driving circumstances affecting the pathology and identification of *Enterococcus cecorum*, an emerging pathogenic bacterium causing musculoskeletal bone problems in broiler chickens. Immunity and the physiological condition of both *E. cecorum* and the broiler chickens as hosts will provide insights into disease comprehension and the reduction in the risk of musculoskeletal bone disease in broiler production.

## 1. Introduction

Bacterial chondronecrosis with osteomyelitis (BCO) is the leading cause of lameness in poultry and has a high impact on birds’ welfare, substandard meat quality, and reduced production [1,2]. This bacterial disease is responsible for high mortality, bird culling, and carcass condemnations due to quality depletion for human consumption, with around 19% of cases being moderate to severe. BCO lameness has been regarded as a significant disease in commercial chicken-meat-producing industries worldwide [3,4,5,6,7]. Vertebral Osteomyelitis (VO), the condition of posterior skeletal and hind limb immobility disorder as a part of spinal cord compression, is a case of lameness that leads to a significant increase in mortality up to 15% [3,4,8,9]. One of the major causal agents of VO is *E. cecorum*, referred to as enterococcal-VO or Enterococcal spondylitis, which differentiates it from the non-bacterial “kinky-back” posture (spondylolisthesis) [10]. VO usually occurs during the 5th to 7th week of gestation and may also cause early embryonic death [11,12]. VO is often associated with infection by a single species or several bacteria, with the most prevalent bacteria belonging to the *Enterococcus* genus [13].

As an emerging infectious disease in broilers, enterococcal-VO has been a concern for researchers and poultry producers in several countries [3,14,15,16], generating studies that identified *E. cecorum* as the predominant bacterium in vertebral lesions in birds with VO [8,14,17], as well as femoral head necrosis (FHN), joint arthritis, and synovitis [4,15,18]. Moreover, *E. cecorum*, which was once considered commensal in animals, has also been implicated in worsening medical problems in humans [19,20,21,22]. Different strains of *E. cecorum* have been isolated and analyzed from various organs, revealing distinct characteristics and raising questions about how disease pathogenesis is initiated, as well as the ideal conditions for *E. cecorum* to thrive and cause infection in different tissues. This review will focus on the factors affecting the prevalence of enterococcal-VO in broiler chickens, as well as the features of both the hosts and bacteria determining disease pathogenesis.

## 2. Methods

The search for relevant studies was conducted using electronic databases, specifically PubMed and Web of Science. Several keywords used in this study cover the correlation between Vertebral Osteomyelitis and *Enterococcus cecorum*, such as “Chicken AND Vertebral Osteomyelitis”, “Chicken AND Spondylitis”, “Chicken AND Kinky Back”, “*Enterococcus cecorum*”, “*Enterococcus cecorum* AND Poultry”, and “*Enterococcus cecorum* AND Broiler”. Some keywords are used to select the genetic factors, such as “Genetics AND *Enterococcus cecorum*”, “Genetics AND *Enterococcus*”, “Gene AND *Enterococcus cecorum*”, and “Gene OR Genetics AND *Enterococcus*”. The keywords such as “Environment AND Enterococcus AND Poultry”, “*Enterococcus cecorum* environment”, “*Enterococcus cecorum* infection”, “*Enterococcus cecorum* culture”, “*Enterococcus* environment”, and “Environment *Enterococcus* poultry” were used to extract information about the presence of *Enterococcus cecorum* in Poultry and the favorable environmental conditions for presence and infection. The other keywords are also employed to support the previously mentioned keywords, such as “Pathogenicity AND Vertebral osteomyelitis”, “Virulence AND *Enterococcus*”, and “Resistance AND *Enterococcus*”. In order to address broiler chickens’ genetics base, we use keywords “Broiler chicken genes” and “Broiler chicken genetics” and then select the articles based on the broiler chicken’s genetic bases related to health and diseases. From the search results, articles were selected based on their titles and abstracts, type of paper, object of study, year of publication, and causal agent of VO that was prevalent in the study. Further details were explored in some articles as needed.

## 3. The Etiology of Vertebral Osteomyelitis in Broiler

Owing to intense genetic selection over the past several decades toward efficient feed conversion, rapid growth rate, and muscling, as well as heavier body weights, the modern broiler has quadrupled in size while consuming significantly less feed over the same growing period [23]. However, this drastic trend has brought about significant drawbacks to the bird’s physiological health over the years, particularly in terms of musculoskeletal diseases and deficiencies [10,24,25]. As briefly introduced, BCO and its associated lameness are an increasingly common disease with significant economic importance and dire implications for the animal’s well-being. BCO lameness is predominantly postulated to be initiated by microfractures induced via mechanical stress on the immature skeletal system of the modern broiler—which is far outpaced by its weight gain [10,26,27,28]. Long chondrocyte columns found in the growth plate of proximal tibial/femoral physis and epiphysis are especially susceptible to such physical stress, often resulting in the formation of osteochondrotic clefts and exposure of the collagen matrix, which leads to transection of blood vessels and eventual necrosis [10]. At the same time, stress from the intensive growing environment further compromises the bird’s immune system, causing translocation of pathogenic bacteria—whether acquired through the environment or internally—into the bloodstream, leading to bacteremia and eventual infection of necrotic sites through binding to nearby exposed collagen matrix, which manifests in clinical lameness signs. 

**Figure 1:**
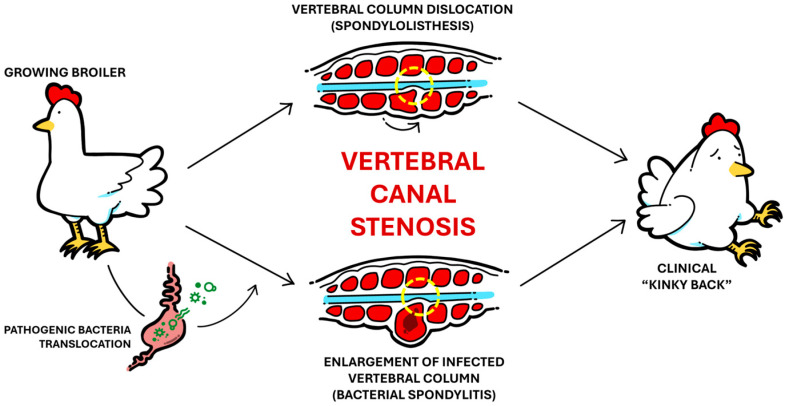
Simple illustrated schematic showcasing the development of non-bacterial spondylolisthesis versus bacterial spondylitis and the clinically identical outcome.

Sharing much similarity with BCO etiology, VO is mostly caused by damage to the vertebral column due to routine involuntary movement, particularly in the free thoracic vertebra (FTV) region [10]. Most commonly referred to in the industry as “kinky-back”, VO causes spinal cord compression due to stenosis of the vertebral canal with the same clinical outcome, though the underlying causes can be vastly different in nature, making a “kinky-back” diagnosis somewhat of a misnomer in production terms. In cases of nonbacterial VO (spondylolisthesis), the broiler’s vertebral column (between T4 and T7) is dislocated, causing physical stenotic compression of the vertebral canal due to kyphotic angulation of the column [29,30]. On the other hand, bacterial VO (bacterial spondylitis) is etiologically similar to the proposed postulation of BCO pathogenesis, with infection of microfractures formed within the vertebral column. Consequently, inflammation and a fibrinous, exudative capsule form within the column, which increases its size and causes similar stenosis of the vertebral canal. In both cases, the resultant clinical signs are practically identical: affected broilers are often underweight and observed in a paraplegic hock-sitting posture, with legs further extended with increasing severity [31]. Figure 1 summarizes this relationship between the two conditions and their clinically identical outcome. 

There is no effective cure or treatment for birds with spondylolisthesis/spondylitis, and culling of affected animals remains the sole effective solution for farmers and producers [32]. Due to this extreme similarity in clinical signs, accurate diagnosis of the underlying cause can potentially be very difficult without subsequent confirmatory necropsy and microbiological evaluation of affected birds, which may contribute to its underreporting from farmers, even though it is currently regarded as an emerging disease of great industrial concern [31]. As such, there has been increased interest in bacterial VO research in recent years from the poultry industry, especially with regard to *E. cecorum* as the etiological agent of interest, particularly during the hatching phase of production [33].

With regard to osteomyelitis, earlier publications have indeed reported the presence of predominantly *Enterococcus* species from Vertebral Osteomyelitis (VO) lesions [5,12], with most reports associating VO with *E. cecorum* infection [5,15,17,29] and increasing prevalence in some cases [3], while some recognize distinct species of *Enterococcus* spp., either solely or with co-infection of *Escherichia coli*, *Staphylococcus* spp., and other *Enterococcus* species [12,30,31]. However, recently published surveillance data on *E. cecorum* incidence in VO cases are somewhat limited outside of industrial communications and independent internal research.

## 4. Characteristics of Enterococcal Bone Diseases in Poultry

Enterococci are a ubiquitous group of bacterial species associated with humans, animals, plants, and the environment. There are at least 63 defined species of *Enterococcus* spp., with a number of entries stated as “candidatus” [34]. While some enterococci strains are commensal in poultry, others negatively affect poultry production, with *E. faecalis* and *E. cecorum* currently being the highest reported cases in poultry [3] as both can induce VO in broiler chickens [13,31,35]. Of note, the rising incidence of *E. cecorum* has been significant since its initial identification [36].

*E. cecorum* has been identified as one of the most frequently present bacteria in osteomyelitis lesions, although it can also be detected in various problems in poultry [3,9]. In the incidence of Femoral Head Necrosis (FHN), the presence of *E. cecorum* can also be identified concurrently with other species [4,37] such as *E. faecalis* and *Enterococcus* spp., which are genetically distinct but can infect birds and cause similar signs of lameness.

**Figure 2:**
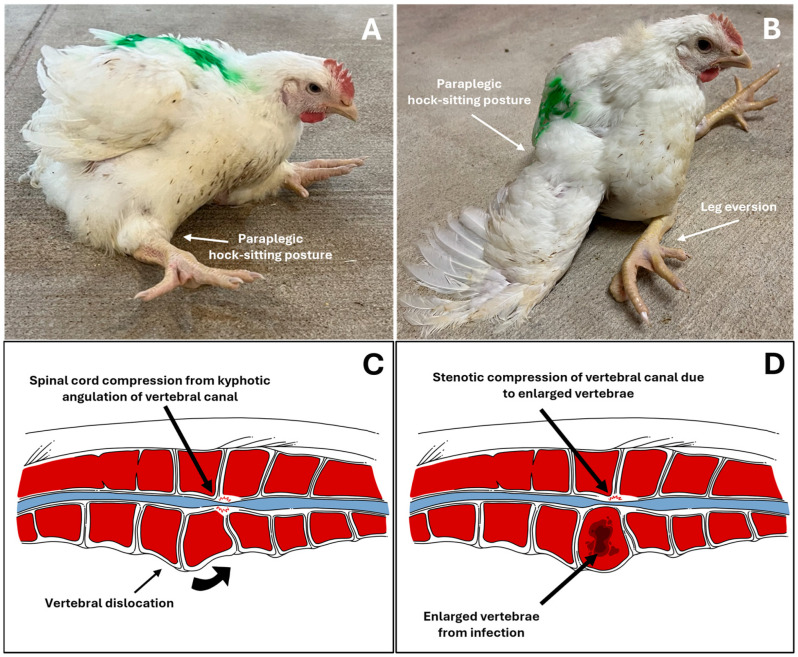
(**A**,**B**): Broiler chickens exhibiting “kinky back” clinical signs, with common paraplegic hock-sitting posture (**A**) and severe cases with extended leg eversion (**B**). (**C**,**D**): Illustrated diagonal cross-section of the thoracic vertebra in non-bacterial spondylolisthesis and bacterial spondylitis, respectively. Vertebral dislocation causing kyphotic angulation of the vertebral column and subsequent stenotic compression of the vertebral canal (**C**); bacterial infection causing gross necrotic lesion with fibrinous exudate capsule within vertebral column, enlarging it and causing stenosis of vertebral canal (**D**). Adapted and modified from Anthney et al. (2024) with permission [1].

VO occurs after infection spreads into the Free Thoracic Vertebral (FTV) bone, particularly causing lesion formation in the fourth thoracic vertebra. This results in the atypical hock-sitting posture with legs extended forward, as shown in Figure 2A,B, and the spinal cord condition illustrated in Figure 2D. As previously mentioned, both bacterial and non-bacterial vertebral lameness display a similar-to-identical hock-sitting posture, greatly adding to the difficulty in diagnosis. Different species of *Enterococcus* have been discovered from the same lesion source of osteomyelitis, including *E. cecorum*, *E. faecalis, E. hirae*, or *E. raffinosus*, or a mixture of these bacteria, for which their presence is the evident cause of VO in poultry [38].

Prior to extensive research into VO pathology, *E. cecorum* was originally known as a commensal strain of gut and cecal bacteria, raising the suspicion that there are differences between commensal and pathogenic strains of *E. cecorum*. The virulence factors among different strains are distinct, especially between isolates obtained from various locations [39].

### 4.1. Initiation of Infection and Clinical Signs

The oro–fecal route is the main portal of entry of *E. cecorum* [40], and secondarily, the other is by inhalation of droplets or aerosols containing the bacteria [5]. Findings through the embryo lethality assay (ELA) have also hinted at the possibility of vertical transmission. Embryos infected with commensal and pathogenic strains of *E. cecorum* exhibit contrasting mortality levels, indicating the existence of strain-dependent factors that influence the rate of death in birds and the occurrence of cases [41]. Severe signs occurred after inoculation, leading to embryonic death, with a significant mortality rate reaching 100% for the pathogenic strains, which can be as low as 0% for the commensal strains of *E. cecorum* [12,42,43,44]. In contrast, some earlier publications indicate higher mortality rates in commensal strain treatment than pathogenic strain; however, the embryo of pathogenic strain treatment shows sepsis that is not present in the commensal strain-treated egg [11].

Pathogenic strains were found to be stronger against lysozyme in the eggs, suggesting the possibility of vertical transmission; however, this remains inconclusive in the current literature. It has been documented that the strains associated with diseased chickens are genetically more diverse than those from their parents, indicating multiple sources of infection [37]. Additionally, *E. cecorum* could not be recovered from the albumen of inoculated eggs after incubation, presumably due to various proteins in the egg environment that inhibited its survival [44].

Pathogenic *E. cecorum* strains colonize the chicken intestinal tracts as early as the first week of life, outcompeting commensal strains that typically emerge by the third week [39]. It is hypothesized that the VO-related intestinal bacteria in BCO lesions have migrated through the intestinal epithelium and dispersed throughout the body, reaching the skeletal organs [5,17,42]. Following the colonization of the host’s gut, multiple factors support *E. cecorum* translocating into the bloodstream (bacteremia), dispersing and infecting various tissues and organs (septicemia), including the spleen, liver, heart, and finally the joint and skeletal system. Prior to the exhibition of clinical signs associated with lameness and paralysis, systemic *E. cecorum* infections can appear nonspecific, including depression, lethargy, and agitated feathers. Once the infection has reached the skeletal system, particularly the FTV, specific signs would appear, such as the “kinky back” posture in broiler chickens [39,45].

### 4.2. Identification of Enterococcus cecorum

*E. cecorum* can be mainly isolated from the infected bone and joint tissues (FTV, tibial, and femoral head), as well as other tissues (spleen, liver, and pericardium) [37]. Traditionally, as the viability of *E. cecorum* declines over time, so too does the possibility of successful isolate recovery from environmental samples (litter, dust, PVC, and other surfaces), although these prospects have become more possible depending on time, environmental sources, and the isolation medium [45,46,47]. A microaerophilic condition with 5% CO_2_ has been suggested for optimal isolation and identification of *E. cecorum* [39]. An automatic genomic annotation of *E. cecorum* isolates obtained from chicken caecum revealed it to be microaerophilic [48]. In addition, the use of selective media, such as blood agar, nalidixic acid-supplemented agar, and chromogenic agar, can further help ease its characterization. In blood agar or medium supplemented with lab-grade blood, *E. cecorum* forms a slight appearance of α-hemolysis with a greenish-brown color spread around cream to grey-colored, mucoid-like colonies [6,49], similar to the appearance of most *Streptococcus* spp. on the same medium [6,49]. Some strains of *E. cecorum* may appear as a small-colony variant [33]. Commercially available chromogenic agar media are often used to identify *Enterococcus* spp. colonies based on a certain color (such as turquoise blue), but they do not differentiate between *Enterococcus* groups. Alternatively, a medium composed of a base agar medium for blood agar (sans blood), supplemented with a β-glucuronidase substrate (5-bromo-4-chloro-3-indolyl-β-D-glucuronic acid) along with colistin-nalidixic acid (CNA), generates turquoise or blue-green *E. cecorum* colonies, whereas *E. faecalis* and *E. faecium* colonies show no color [47]. A Todd Hewitt Broth with 1% yeast extract (THBY) or Tryptic Soy Broth can also be used as the broth medium [49].

*E. cecorum*’s cell wall properties, like those of other Gram-positive bacteria, are thick and molded with layers of peptidoglycans [50]. Lysozyme and proteinase K have been applied to facilitate the destruction of tough *E. cecorum* peptidoglycan molecules right before the DNA extraction phase [42,51]. However, together with *E. faecalis*, pathogenic *E. cecorum* was discovered to be extra resistant to lysozyme compared to the non-pathogenic strains [44].

Current molecular identification of *E. cecorum* involves different methodologies, such as PFGE (Pulsed-Field Gel Electrophoresis), PCR (Polymerase Chain Reaction), 16S rRNA partial genome sequencing, and WGS (Whole-Genome Sequencing) [6,14,51,52,53,54]. A set of multiplex PCR markers was developed to distinguish *E. cecorum*, and they were tested for their specificity on conserved regions specific to *E. cecorum* and pathogenic strains [49]. The *SodA* gene, which encodes superoxide dismutase, has been identified as an effective marker for distinguishing between *Enterococcus* species and has been developed as a biomarker to detect *E. cecorum*. The combination of marker *CpsO* and *SodA* genes in a multiplex PCR test has thus become a powerful tool for distinguishing between the pathogenic and non-pathogenic strains of *E. cecorum* [49].

## 5. Internal Factors of *Enterococcus cecorum* Infection

Biological features, including genetic and physiological characteristics of *E. cecorum*, may define its infection. Studies have highlighted significant differences between pathogenic and commensal strains of *E. cecorum* in terms of these features.

### 5.1. Genetic Characteristics of Pathogenic and Commensal EC

Through genomic sequencing and various methods of genetic analysis, researchers have identified genetic characteristics to distinguish between commensal and pathogenic strains of *E. cecorum* and determined genetic factors that contribute to its virulence. The pathogenic strains are specifically obtained from non-intestinal organs (vertebral lesions, pericardium, femoral heads, and tibial heads), while the commensal strains were generally isolated from intestinal organs (caecum and colon) [4,14,42]. Different studies have targeted genetic characteristics, such as genes for resistance (resistome), elements of “jumping genes” (mobilome), and genes responsible for increasing virulence (virulome) for structural and functional analysis [38,42,51,55]. Besides the development of microbes’ potential to transfer to different organisms and sites, genetic diversity also broadens virulence, supporting their rapid pathogenicity [56,57]. Interestingly, Enterococci’s wider pangenome can also facilitate its environmental plasticity and ability to adapt to various niches [58].

The pathogenicity of *E. cecorum* can be supported substantially by the presence of resistance properties in its genome, known as the resistome, which enhances its survival abilities against various antimicrobial treatments [55,59]. Multiple mutations are identified in the Antimicrobial Resistance Genes (ARGs) possessed by *E. cecorum*, either in commensal or pathogenic strains [60]. The use of antibiotics may induce the emergence of ARGs across the bacterial population, especially after antibiotic treatment [61]. For instance, the successful counteraction of enrofloxacin and ampicillin by certain strains of *E. cecorum* is due to the presence of single-nucleotide mutations in the genes [55]. The combination of lincomycin and spectinomycin in the early developmental stage of broilers could prevent the diseases caused by *E. cecorum* and reduce the load in the ceca. Still, bacteria with resistance genes may continue to grow significantly even after treatment [61]. Therefore, resistance tends to be present in both commensal and pathogenic bacteria [55,62], with the commensal strains bearing more resistance genes and being distinct from the patterns of the pathogenic strains. This contrasts with earlier research, which shows resistant genes being more prevalent in the pathogenic than the commensal strain of *E. cecorum* [63]. These findings highlight the need for further research to elucidate the underlying modes of action between commensal strains and pathogenic ones found in lesions.

*VanA*, a major gene associated with vancomycin resistance, encodes the altered structure of peptidoglycan for D-lactate termination [7,58]. *VanA* is one of the gene targets for recognizing medically significant vancomycin-resistant Enterococcus (VRE) strains, as vancomycin is widely considered among the last-resort antibiotics. The *VanA* gene is relevant to resistance against both vancomycin and teicoplanin. Susceptibility to teicoplanin while being resistant to vancomycin is associated with the *VanB* phenotype-*VanA* genotype [7]. Huang et al. (2024) [55] found that Vancomycin-resistant genes were only found in non-clinical strains of *E. cecorum*. Its presence as an operon-containing gene cluster enables the strain to gain higher adaptability [7,54,55]. Moreover, *VanA* is often found in the downstream of a mobile element, making it possible to transfer between species and populations [38,43,54].

A group of genes responsible for horizontal gene transfer, or enabling evasion of the host’s guard, is defined as the mobilome. It comprises genes identified as mobile genetic elements (MGEs), which include transposases, integrons, plasmid elements, phage, and other similar elements. Different sets of genomic analysis identified an intact phage element along the span of the *E. cecorum* genome, but without virulence factors within the phage [38,51]. Two plasmid structures were found in the genome of *E. cecorum*, one of which is shared between the Enterococcal species [38]. More importantly, the other genes underlying bacterial protection against antibiotics can often “move and jump” due to upstream MGEs [7,54,55].

The presence of virulomes in the genome of pathogenic *E. cecorum* is functionally most likely to be the determinant of pathogenicity in these strains. The genes commonly used to detect virulence of *Enterococcus* spp., such as *efaA*, *gelE*, *ace*, *eeP*, and *asa1*, do not reveal an exact pattern of virulome in the pathogenic strains of *E. cecorum* [64]. These virulence patterns, although more prevalent in the pathogenic strains, may exist in strains originating from the intestines or ceca of healthy birds. Rather than only using the classic virulence genes of *Enterococcus* spp., the virulome of *E. cecorum* exhibits a more complex pattern that is multifactorial in nature and involves a broader set of genetic patterns and another clade-specific gene. Nevertheless, another study on several strains of *E. cecorum* has revealed more conserved regions among the genomes of pathogenic strains compared to commensal ones [65]. Certain virulence patterns have been consistently identified in pathogenic strains, such as the *CpSO* gene (encoding for the capsular synthesis locus) [65], and have been used as a marker for multiplex PCR to detect pathogenic *E. cecorum* [49]. Laurentie et al. (2023) concluded six genetic patterns to differentiate 94% of the *E. cecorum—*causing disease [54]. Rhoads et al. (2024), using the more meticulous category of pathogenic and non-phenotypic strains, investigated whether the increasing virulence in sepsis and BCO strains resulted from horizontal transfer between bacteria or by mutation [51]. The pathogenic groups and non-phenotypic *E. cecorum* strains tend to be divided into clades with different virulence factors persisting between them [38,51], suggesting that gene alteration is a more plausible scenario for the formation of increasing virulence patterns rather than horizontal gene transfer [38,51]. On the other hand, around 47% and 42% of similarities were observed between coding and uncharacterized genes, respectively, in several isolates of *E. cecorum*, indicating horizontal gene transfer between groups [54].

Finally, mutations in *PEG231* (protein for serine aminopeptidase S33), *EIIABC* (component for PTS fructose-specific EIIABC), *CBCL1* (a ligase enzyme of 4-chlorobenzoate—CoA), *ElaA* (an N-Acetyltransferase enzyme in GNAT family), *EttA* (a protein of energy-dependent translational throttle), *RpoN* (factor for RNA polymerase), and *YheH* (a putative resistance gene for ABC transporter ATP-binding/permease protein) were considered as the key to the adaptability of *E. cecorum* with respect to its virulence. These genes are also conserved across different pathogenic bacteria and reveal the supporting functions in each of the infections [51].

### 5.2. Physiological Features of Enterococcus cecorum

Genetic and physiological characteristics are inextricably linked in the analysis of *E. cecorum*. While genetic analysis provides valuable insights into the virulence factors of *E. cecorum*, it often represents only the potential traits possessed by the gene structure. Biochemical tests are frequently conducted to complete the differentiation of the bacteria between genera, species, and clades or strains. For example, unlike most *Enterococcus*, all—or at least most–pathogenic *E. cecorum* strains are unable to ferment mannitol. This inability of pathogenic *E. cecorum* to ferment mannitol is due to the absence of the mannitol enzyme gene orthologs, which are conserved in the commensal *E. cecorum* [65]. The failure of certain *E. cecorum* strains to metabolize mannitol can thus be used to discriminate the pathogenic type of *E. cecorum* [41,66]. The lysozyme-resistant strains were known to lack the ability to process mannitol [41,66]. With this in consideration, certain biochemical and physiological characteristics can be used to distinguish between *E. cecorum* isolated from lesions and ceca, greatly enhancing *E. cecorum* differentiation (Table 1).

*Enterococcus* spp. commonly produce hemolysin and enterocin, as is the case with *E. cecorum* [58]. Hemolysin can potentially damage the host’s cells, while enterocin helps to control their environmental dominance by inhibiting the growth of at least one type of bacteria. Its ability to perform hemolysis and the presence of enterocin have been believed to support its pathogenic potential [67], along with other acquired virulence factors.

## 6. External Factors of *Enterococcus cecorum* Pathogenicity

Multiple external factors, including the immediate surrounding environment and host factors, determine the initiation, susceptibility, and development of *E. cecorum*.

### 6.1. Environmental Factors

Associations of lameness with hock burns, footpad dermatitis, and bird cleanliness suggest that a suboptimal physical environment (e.g., litter and air quality) may be detrimental to leg health [2]. As it is labeled an opportunistic pathogen, *E. cecorum*—with the acquired virulence factors—can be translocated from its usual environment as part of the gut to the bloodstream through the leakage of the gut barrier, causing bacteremia. Environmental stress can also induce and exacerbate bacteremia. Hyperthermal conditions appear to significantly reduce the tight junctions’ mRNA expression, affecting their integrity and thereby increasing the severity of *E. cecorum* infection [68]. Additionally, environmental heat stress may negatively affect birds’ immunity through local and systemic inflammatory response [69]. Inflammatory immune responses, such as production of inflammatory leukocytes, are not increased under heat stress and thus reduce the bird’s immunity against bacterial infections [69]. Interestingly, cold stress stimulation in early stages, on the other hand, can potentially direct various immune factors to protect the broiler for a forthcoming acute cold stress at a later stage [70]. As another environmental factor crucial to broiler stress and welfare management, stocking density is another aspect that may significantly impact bird susceptibility to infections, whether through stress, activity levels, or injuries [71]. Lower flock density can support the locomotive ability of the broiler regardless of the growth rate [72], while increased individual space has been shown to improve the health of the birds’ microbiome [73].

Beyond the host’s body, temperature also affects *E. cecorum* tenacity. The bacteria are able to outlive and optimally grow in lower temperatures (15 °C) with 32% relative humidity (RH) in dust, litter, and polyvinyl chloride, but they cannot endure longer than one hour in higher temperatures (65 °C and 70 °C) [6,46]. Compared to the other enterococci, *E. cecorum* shows limited halotolerance, as evidenced by its limited growth in 6.5% NaCl [6].

The identification of *E. cecorum* in concrete, dust, feces, and equipment confirmed its presence, with phenotypic similarities to *E. cecorum* isolated from chicken vertebrae, indicating that *E. cecorum* can survive on different surfaces in the environment [16,74]. For instance, on the surface of concrete, *E. cecorum* may remain for at least 21 days [45]. Additionally, farms with a history of VO outbreaks tend to experience subsequent infections in the flock due to *E. cecorum*’s survivability on various surfaces and environmental conditions [75].

Diet composition is another factor that can significantly influence the host’s microbiome community in broiler chickens, either by reducing communities belonging to certain opportunistic pathogenic species (including *Enterococcus* spp.) or by controlling the composition of said microbiome [76]. Contamination with toxic substances, including mycotoxins, during pre- and post-harvest or during the storage of feed has been implicated in damaging the intestinal barrier and progressively increasing lameness cases in broilers [77,78]. On the opposite end, beneficial effects from broiler diet supplementation with minerals have long been documented in the literature. Phosphorus, calcium, and phytase supplementation may help influence positive microbial diversity within the broiler gut microbiome, which can bolster bird performance and health [79] with positive implications regarding protection against infections, such as *E. cecorum*. As another example, inclusion of high-quality organic trace minerals (such as zinc, manganese, and copper) in the broiler diet has been documented to increase expression of tight junction proteins regulating epithelial membrane integrity in the gastrointestinal tract, thereby improving “leaky gut” and decreasing translocation events, which directly correlates to decreased infection rates and the ensuing lameness [24].

### 6.2. Host Factors

While an embryo may be able to endure infection in ovo, *E. cecorum* can survive and cause an impending infection in organs in the future [12]. The immune factor is the focal point of the host’s factor. Once pathogenic *E. cecorum* invades the host, an interplay occurs between the host’s immunity agents and the pathogen’s ability to confront the system. As bacteremia occurs, the first line of the host’s protection has already been compromised. Other triggers may follow, such as necrotic enteritis (NE) caused by *Clostridium perfringens* toxins types A and G, which may induce intestinal barrier leakage and intensify *E. cecorum* growth [8,80]. Furthermore, NE can also be induced by heat stress, which has been shown to negatively affect broiler chickens’ IgA, IgM, and IgY serum levels, intestinal barrier, and germinal centre (GC) secretion, leading to potentially reduced vaccination efficacy and protective effects against infections [81].

Extensive feeding might also increase the growth rate of *E. cecorum*, which can also lead to infection [82]. Physiological issues, such as elevated blood glucose levels, have been shown to increase the bacterial count of *E. cecorum* in the liver and thoracic vertebrae tissues, where the infection is localized, thereby exacerbating disease progression. Increasing blood sugar levels have been indicated as a challenge to immunity [83]. Feed or diet may also contribute to downregulation or upregulation of genes related to environmental stress. The expression level of heat shock protein-70 (*HSP-70*) will increase in the liver, hypothalamus, and muscles of chickens during heat stress, and it can be either upregulated and downregulated by various diets and supplements such as leptin [84], trace minerals [85], merulenin [86], and ascorbic acid [87].

Concurrent diseases in the intestines of broiler chickens could affect the subsequent *E. cecorum* infection differently. Although *E. cecorum* infections tend to happen simultaneously or after another infection has occurred in the host, interactions with other organisms may generate different types of responses. For example, a prior infection with immunosuppressive organisms, particularly viruses such as reovirus and avian viruses, could increase the prevalence of *E. cecorum*-induced lesions [4,88], impact higher positive *E. cecorum* reisolation, and increase mortality after co-infection tests with chicken anaemia virus (CAV) [88]. Thus, it stands to reason that other avian viruses with similar immunosuppressive outcome may potentially worsen the severity of *E. cecorum* infections, such as Marek’s disease [89,90], infectious bursal disease (IBD) [91,92], and fowl adenovirus (FAdv) [93]—whether such viruses have been examined in concurrent infection with *E. cecorum* or not. In contrast, however, co-infection with coccidian parasites may provoke immunosuppression for *E. cecorum* infections, thereby paradoxically preventing its bacteremia and reducing VO lesions [91,94]. Additionally, different immunosuppressive agents, such as mycotoxins, vaccines, or immunosuppressive chemicals like dexamethasone, might generate divergent effects due to different metabolic pathways [88].

Various genes that are directly involved in bone metabolism can also influence susceptibility to bacterial infection in bone tissues. For example, polymorphisms of *TNFRSF11A*, a gene encoding the RANK protein, are significantly related to bone strength, dimensions, and mass in broiler chicken. This gene is associated with many hormones and cytokines and functions in osteoclast cell differentiation, activation, and resorption support [95]. In layers, this gene is also found to significantly impact bone quality together with the other three candidate genes (*SERPINE3*, *INTS6*, and *POSTN*) located in the same region. These genes are predicted to be responsible for protection with respect to fracture, bone quality, osteoblast differentiation, and bone formation, respectively [96], all of which are directly correlated with *E. cecorum* susceptibility in the currently accepted model of pathogenesis and pathophysiological changes.

Finally, in a broader landscape regarding broiler genetics and its impact on bacterial infection susceptibility, there also exist several discussion points that warrant increased attention. Favorable genetic trait selection through extensive breeding programs has resulted in the modern broiler chicken with improved production traits such as high feed efficiency, fast muscling, and weight gain. Compared with the broiler chicken strains from the 1950s, the modern broiler strains grow larger and faster, with noticeable behavioral difference between types. Slow-growing chickens tend to stand and walk more often than the fast-growing strain [97], the latter of which is widely notorious for having higher susceptibility to various locomotive disorders [72]. Interestingly, Chen et al. (2018) found the prevalence uniformity of osteochondrosis lesions in FTV (>70%) among modern broiler strains and 1950s broiler, indicating VO as a common disease regardless of the strains [98]. One possible factor that may explain such a phenomenon lies in the trade-off of bone parameters between past and modern broiler strains. In comparison with the slower-growth chicken strains, while genetic selection has resulted in better overall bone quality in the modern broiler chicken, its bone density is lower compared to chickens with a slower growth rate when the total mass load is taken into consideration [99]. Other findings in the current literature have also revealed a somewhat clouded overview regarding the disconnect between genetic improvement efforts in the modern broiler and certain heritable outcomes related to its limb and skeletal health. A study by Siegel et al. (2019) involving meta-analysis of breeding values from millions of pedigree pureline broilers over a decade-long collection period has found that while certain limb traits have indeed been improved thanks to selection, the trends for others—including femoral head necrosis, a disease closely related to VO—are not quite as apparent, oftentimes pointing to the contrary [100]. Kapell et al. (2025) have also discovered that the genetic correlation between production traits and gait scores is unfavorable, even though traits intended to improve gait score have long been included in breeding programs within the past decades [101]. It is evident that much research remains to be carried out to gain further insights into the true effectiveness of broiler genetics improvement selection programs and their heritability indices in the context of BCO and VO pathogenesis, which might prove a significant challenge to overcome as the private industry may not be readily willing to disclose proprietary information so as to retain a competitive edge.

### 6.3. Potential Treatment

The emergence of *E. cecorum* outbreaks in commercial farms necessitates the need for treatments. Antibiotic use in poultry, such as lincomycin–spectinomycin, has long been a significant concern for the potential antibiotic resistance it can promote—especially at the end of treatment—and the reduction in beneficial bacteria in the ceca [61]. Metaphylactic treatment, or a combination of antibiotics to treat infections including *E. cecorum*, is thus often used as a strategy, especially during the beginning stage of infection [75]. Utilizing organic acids and natural identical compounds, either solely or simultaneously with antibiotics, has been shown to lower *E. cecorum* infection to 100% reduction [102]. According to a 2019 study, certain commercially available strains of *Bacillus* probiotics for poultry are efficient at suppressing pathogenic *E. cecorum* in vitro, even though the results depend on the target strain [103]. Another polyvalent-killed vaccine was created to treat *E. cecorum* infection, but it showed non-significant results when an antibody against *E. cecorum* was established [104]. In terms of genetic selection, there is currently ample room for improvements and discoveries among the pool of beneficial candidate genes contributing to bone health, including those with multi-trait impacts. For example, such candidate genes should include those involved in the digestive efficiency of chickens, which has been shown to be positively correlated with increasing bone strength thanks to increased phosphorus digestion and bioavailability in birds with higher digestive efficiency [105]. Finally, biosecurity has been proposed as the most effective method for preventing horizontal transmission between houses or with respect to the environment, as with many other infectious diseases common to the poultry industry [16,83].

**Figure 3:**
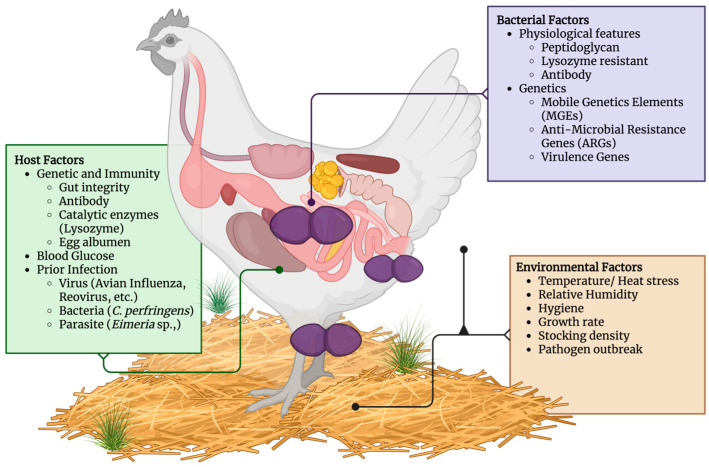
Factors influencing *E. cecorum* infection in broiler chicken (Created in https://BioRender.com).

Figure 3 visualizes all previous discussion points as a summary of the complex interplay between external and internal factors governing *E. cecorum* pathogenicity.

## 7. Conclusions and Future Directions

Transmission of *E. cecorum* between infected flocks and birds has been proposed as the most probable pathway for *E. cecorum* transmission [33,37,40,45,74]. Several factors influence the initiation and development of infection, from infection, bacteremia, and septicemia until *E. cecorum* can invade the FTV. Lack of thorough disinfection following prior outbreaks and weak biosecurity measures may contribute to persistent recurring outbreak events in the same facility due to the long survivability of viable *E. cecorum* on various surfaces [74].

The infection that ultimately causes issues commonly associated with *E. cecorum*, including embryo lethality and skeletal issues (lameness and VO), is strain-dependent. However, the characteristics of both commensal and pathogenic strains are highly variable. Aside from the presence of virulence factors, ARGs do not substantially improve the virulence of the pathogenic strains [51,54,59,60,106]. Instead, several functional genes are reported to be conserved in most pathogenic strains, one of which has been used as a molecular marker for detecting pathogenic *E. cecorum* [55]. Bacteriocin and hemolysin are among the current hypotheses that may explain *E. cecorum*’s capability to be a dominant bacterium in the gut until it successfully colonizes the FTV tissue and causes VO, besides the reduced gut integrity associated with reduced immune functions in the broiler. Once *E. cecorum* enters the gastrointestinal tract, the internal and other environmental factors begin a cascade of interactions that lead to eventual infection. Environmental factors such as heat stress or prior infection by *C. perfringens*, reovirus, and avian influenza virus can induce infection either through the gut’s compromised integrity or an already weakened immune system, leading to *E. cecorum* bacteremia until it reaches the other organs (septicemia), particularly the FTV, and this further causes a distinct case of lameness called enterococcal-VO [88].

With poultry production continuing to be one of the top producers of affordable and high-quality animal protein for a growing world, accelerated research to improve the bird’s health, productivity, and well-being remains an important goal for the poultry industry at large. Evidently, there remain significant knowledge gaps in the current literature surrounding *E. cecorum* etiology and factors governing its pathogenicity in practical settings [107,108]. Future research should therefore emphasize these lacking aspects, particularly external factors (such as the housing and production environment), and novel research should be conducted to determine potential unknown *E. cecorum* virulence genes and factors, such as assay development [16,46,74]. Moreover, there has also been increased interest in detailed research of *E. cecorum* infection and its long-term effects in broiler lameness using induction models with high practicality and translatability, as well as optimal growth conditions under which *E. cecorum* can be reliably cultivated in mass quantities for vaccine development and production in recent years. Finally, due to the clinically identical outcome commonly known as “Kinky-back” [10,32], which has vastly different causes (bacterial spondylitis versus non-bacterial spondylolisthesis), farmers, producers, and the scientific community should promote increased detailed testing and reporting of suspected animals to accurately determine disease prevalence, toward which effective therapeutic treatment and preventive plans can be developed.

## Figures and Tables

**Table 1:** The physiological differences between pathogenic and commensal *E. cecorum* strains.

Features	Commensal	Pathogenic	References
Lysozyme susceptibility	Lysozyme-susceptible, thus lower survival in egg albumen	Lysozyme-resistant, 320 times stronger living in egg albumen	[44,66]
Biofilm formation	Weak–strong	Weak–no biofilm, weak–strong unrepeatable result	[16]
Mannitol Fermentation	Generally possesses the ability to metabolize mannitol	Defect in mannitol metabolism High mortality Reducing weight gain in host	[41]

## Data Availability

Not applicable.

## Abbreviations

The following abbreviations are used in this manuscript:

ARG	Antimicrobial Resistance Gene
BCO	Bacterial Chondronecrosis with Osteomyelitis
C	Celcius
CNA	Colixin-Nalidixic Acid
EC	*Enterococcus cecorum*
FAdv	Fowl Adenovirus
FTV	Free Thoracic Vertebra
IBDV	Infectious Bursal Disease Virus
MGE	Mobile Genetic Element
NDV	Newcastle Disease Virus
NaCl	Natrium Chloride
RH	Relative Humidity
VO	Vertebral Osteomyelitis
